# Reference genome assemblies for the North American bumble bees *Bombus flavifrons* and *Bombus fervidus*, two phenotypically polymorphic species from distinct phylogenetic lineages

**DOI:** 10.1093/g3journal/jkag041

**Published:** 2026-02-17

**Authors:** Jeffrey D Lozier, Rena M Schweizer, Sheina B Sim, Heather M Hines, Michael G Branstetter, Ligia R Benavides, Scott M Geib, Jonathan Berenguer Uhuad Koch

**Affiliations:** Department of Biological Sciences, The University of Alabama, Tuscaloosa, AL 35487, United States; U.S. Department of Agriculture, Agricultural Research Service, Pollinating Insects Biology, Management, Systematics Research Unit, Logan, UT 84341, United States; Division of Biological Sciences, University of Montana, Missoula, MT 59812, United States; U.S. Department of Agriculture, Agricultural Research Service, Daniel K. Inouye U.S. Pacific Basin Agricultural Research Center, Tropical Pest Genetics and Molecular Biology Research Unit, Hilo, HI 96720, United States; Department of Biology, The Pennsylvania State University, 208 Mueller Laboratory, University Park, PA 16802, United States; U.S. Department of Agriculture, Agricultural Research Service, Pollinating Insects Biology, Management, Systematics Research Unit, Logan, UT 84341, United States; U.S. Department of Agriculture, Agricultural Research Service, Pollinating Insects Biology, Management, Systematics Research Unit, Logan, UT 84341, United States; Museum of Comparative Zoology, Harvard University, 26 Oxford Street, Cambridge, MA 02138, United States; U.S. Department of Agriculture, Agricultural Research Service, Daniel K. Inouye U.S. Pacific Basin Agricultural Research Center, Tropical Pest Genetics and Molecular Biology Research Unit, Hilo, HI 96720, United States; U.S. Department of Agriculture, Agricultural Research Service, Pollinating Insects Biology, Management, Systematics Research Unit, Logan, UT 84341, United States; Manoa, Pacific Cooperative Studies Unit, University of Hawaii, 3190 Maile Way, Honolulu, HI 96822-2279, United States

**Keywords:** bumble bees, pollinator, biodiversity, mimicry, color pattern, genome assembly

## Abstract

We present genome assemblies for 2 bumble bee (Hymenoptera: Apidae: *Bombus*) species: *Bombus* (*Pyrobombus*) *flavifrons* and *Bombus* (*Thoracobombus*) *fervidus*. These species are widespread pollinators across North America and both exhibit intraspecific color pattern variation across their ranges due to Müllerian mimicry. These genomes will thus be a useful resource both for studies of native pollinator biology and the evolution of phenotypic variation. We used a combination of single molecule HiFi sequencing and Hi-C sequencing in each species to produce highly contiguous and complete genome assemblies. Utilizing publicly available data, the Eukaryotic Genome Annotation Pipeline was used to generate gene annotations. The *B. flavifrons* genome was assembled into a total of 483 scaffolds, with 18 primary assembled molecules representing putative chromosomes (scaffold N50 = 15.6 Mb), with a total length of 310.5 Mb. Annotation identified 12,476 genes (10,137 protein coding) with a Benchmark of Single-Copy Orthologs (BUSCO) protein score of 97.9% complete (97.6% complete and single copy). The *B. fervidus* genome was assembled into a total of 181 scaffolds, with 19 assembled chromosomes (scaffold N50 = 15.3 Mb, contig N50 = 10.3 Mb), and a total length of 314.3 Mb. Annotation revealed 13,353 genes (10,599 protein coding), with a BUSCO protein score of 98.8% complete (98.3% complete and single copy). We present summaries of gene and repetitive element distributions across the putative chromosome scaffolds and synteny analyses of both species to closely related chromosome-scale *Bombus* genomes.

## Introduction

Bumble bees (Hymenoptera: Apidae, *Bombus* Latreille, 1802) are pollinators of flowering plants in natural and agricultural settings (Thorp et al. 2002; Velthuis and van Doorn 2006). Bumble bees are also of significant interest for ecology and evolutionary biology, with the genus spanning much of the globe, occupying diverse habitats, and exhibiting considerable phenotypic variation both within and among species (Williams 2007; Hines 2008; Woodard et al. 2015). Increasing species-specific genomic resources for bumble bees will thus contribute greatly toward ecological and evolutionary genetics studies of bumble bee diversity, including studies of pollination biology, biogeography, and phenotypic trait variation. Bumble bees have thus been the focus of numerous genome sequencing efforts, with 48 of the ∼265 species globally now having genome assemblies in the National Center for Biotechnology Information (NCBI) Genomes dataset at the time of this writing (accessed 2025 July 21), although only 12 species have Reference Sequence Database (RefSeq) level annotations.

One of the most captivating characteristics of bumble bees is the tremendous diversity in colorful pigmentation of their dense cuticular setae (hairs) associated with different body segments. Bumble bees exhibit more than 400 different recorded color patterns (Williams 2007; Rapti et al. 2014; Ezray et al. 2019). The bright color patterns of these stinging insects represent an example of Müllerian mimicry, where harmful species converge on similar phenotypes in geographic sympatry as a shared warning signal to common predators. There are at least 24 of these distinct mimicry complexes globally (Williams 2007). In North America, the ∼50 species broadly group into 3 major mimicry groups associated with Pacific Coastal (black with yellow stripes), Rocky Mountain (similar to Pacific patterns but with ferruginous, or reddish abdominal stripes), and Eastern (anterior yellow and posteriorly black) regions, although there can be substantial transition zones between these regions and some species or populations exhibit intermediate coloration (Lozier et al. 2016; Ezray et al. 2019). Importantly, some species are polymorphic, and populations in different parts of a species range may diverge phenotypically to match the locally dominant color pattern. Interspecific and intraspecific color pattern variation is not unique to particular bumble bee clades but extends throughout the bumble bee phylogeny, with species exhibiting population-level variation found across distantly related subgenera (Ezray et al. 2019). Reference genomes for species exhibiting color polymorphism from across the *Bombus* phylogeny will thus contribute toward understanding the genetic basis of mimicry in bumbles and aid the development of the genus as a new model for the study of phenotypic radiations (Hines and Rahman 2019; Tian et al. 2019; Heraghty et al. 2020; Sun et al. 2021).

**Fig. 1.**
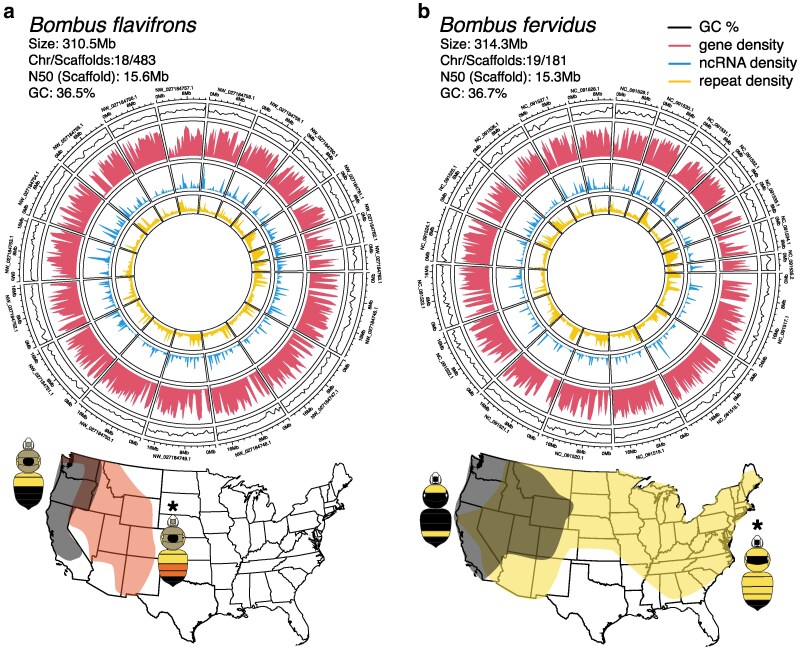
Summaries of genome features (top) and depiction of major color pattern distributions (bottom) for a) *B. flavifrons* and b) *B. fervidus*. Outer rings represent the pseudomolecules identified as putative chromosomes with RefSeq scaffold identifiers and size (Mb), with inner rings (in order) representing the GC%, protein coding gene density, ncRNA density, and RepeatModeler repetitive element density (all calculated per 500 Mb window). Maps show approximate outline distributions of color forms in the conterminous USA (form used for genomes marked with *), depicting only the extreme forms for each species (ie no intermediates) and the *B. fervidus* map including both *B. fervidus* and *B. californicus*, which have overlapping color patterns (see Koch et al. 2018 for details).

*Bombus flavifrons* (Cresson) and *Bombus fervidus* (Fabricius) represent 2 species that exhibit color pattern polymorphism throughout their ranges (Fig. 1). *Bombus flavifrons* belongs to the subgenus *Pyrobombus*, which is in the major “short-faced” clade of bumble bees, while *B. fervidus* belongs to *Thoracobombus* subgenus, which falls in the “long-faced” clade and thus represent polymorphic species from lineages that diverged ∼24 million years before present (Hines 2008; Williams et al. 2008). *Bombus flavifrons* occupies mid-elevation regions of western North America, ranging from the Pacific Coast to the Rocky Mountains, where they range from yellow and black banding pattern on abdominal segments 1 to 4 in the west to yellow and red banding patterns in the east, following the expected spatial transitions in major color pattern complexes (Ezray et al. 2019). *Bombus fervidus* is somewhat more complicated. The species is wide-ranging from the east to west coast across North America, with populations exhibiting primarily yellow abdominal coloration patterns, but occasionally exhibiting black abdominal forms when located in the western Pacific mimicry zone (Koch et al. 2018). Interestingly, *B. fervidus* has a genetically distinct sister species, *B. californicus* Smith, which exhibits similar polymorphism as *B. fervidus* but is restricted to western North America (Koch et al. 2018). Polymorphisms in *B. fervidus* and *B. californicus*, with both species showing the same convergence to local mimicry patterns, has led to historical taxonomic confusion with regard to their species status (Williams 1998) as well as questions regarding the evolution of pigment variation. A genome assembly for *B. fervidus* will ultimately help address these issues.

We report new annotated reference genomes for *B. flavifrons* and *B. fervidus* that contain near chromosome-scale scaffolds assembled using long-read Pacific Biosystems (PacBio) HiFi and short-read element Aviti Hi-C sequencing. The *B. flavifrons* genome adds to the growing number of assemblies in the *Pyrobombus* subgenus, while *B. fervidus* represents one of the first annotated reference sequences for the *Thoracobombus* in the USA. Expanding genome resources for these taxa will enable further genomic and transcriptomic analysis to uncover the basis of trait variation, study evolutionary history, investigate species status, and understand the nature of adaptive evolution within these phenotypically polymorphic species across their ranges and across the *Bombus* phylogeny.

## Methods

### Samples used for sequencing

Samples of *B. flavifrons* and *B. fervidus* were collected by J.B.U.K. in Utah, USA. One *B. flavifrons* gyne (diploid female, red form; Fig. 1a) was collected at Butterfly Lake in the Uinta Mountains (40.721°N, 110.868°W, 3,160 m) on 2021 August 14. One *B. fervidus* drone (haploid male, typical “*fervidus*” yellow form; Fig. 1b) was collected at the Utah State University Research Greenhouse in Logan, UT (41.758°N, 111.812°W, 1,411 m) on 2021 August 13. Sample information was submitted to NCBI for *B. flavifrons* (BioSample: SAMN39995642; Sample name: Bombus_flavifrons_JBK064_abdomen; BioProject PRJNA1078337; Assembly: iyBomFlav1_principal) and *B. fervidus* (BioSample: SAMN40263623; Sample name: Bombus fervidus_BK054_abdomen; BioProject: PRJNA1083964; Assembly: iyBomFerv1). Samples were frozen in liquid nitrogen and stored at −80 °C until transport on dry ice to the United States Department of Agriculture-Agricultural Research Service (USDA-ARS) Pacific Basin Agricultural Research Center in Hilo, Hawaii, USA for genomic data generation.

### HiFi and Hi-C sequencing

Sequencing methods are similar to those used for the recent assembly of *Bombus huntii* Green (Koch et al. 2024) and *B. pensylvanicus* (De Geer) (Lozier et al. 2025) and generally follow the methods being used for the Beenome100 project (https://www.beenome100.org/), a United States Department of Agriculture-led initiative to assemble and annotate genomes from native U.S. bee species. Genomic DNA was isolated from abdominal tissue for each sample using the Qiagen MagAttract HMW DNA Kit (Qiagen, Hilden, Germany) fresh or frozen tissue protocol and purified using 2:1 polyethylene glycol with solid-phase reversible immobilization beads (DeAngelis et al. 1995). Abdominal tissue was used because off-target reads (eg microbial DNA) are of interest for other aspects of the Beenome100 project. DNA was quantified using a dsDNA broad range Qubit assay (Thermo Fisher Scientific, Waltham, Massachusetts, USA) and the fluorometry function of a DS-11 Spectrophotometer and Fluorometer (DeNovix Inc, Wilmington, Delaware, USA), and purity was determined using OD 260/230 and 260/280 ratios from the DS-11's UV–Vis spectrometer feature.

For PacBio HiFi library generation, DNA was sheared to a mean size of 15 to 20 kb with a Diagenode Megaruptor 2 (Denville, New Jersey, USA). We prepared a single SMRTbell library from DNA for each species using the SMRTbell 3.0 library preparation kit following manufacturers protocols for low-input samples (Pacific Biosciences, Menlo Park, California, USA) and then used Ampure PB beads (Pacific Biosciences) to remove <3 kb fragments. Libraries were quantified using Qubit HS (high-sensitivity) dsDNA reagents and sized on an Agilent Fragment Analyzer (Agilent Technologies, Santa Clara, California, USA) using a HS large fragment kit to determine molar concentration. The libraries were each bound for sequencing and sequenced on an 8 M Single-Molecule, Real-Time (SMRT) cell on a PacBio Sequel IIe instrument using default parameters and set to output HiFi reads.

An Arima Hi-C kit was used to create Hi-C libraries (Arima Genomics, San Diego, California, USA) using cross-linked thorax tissue samples from the same individuals used for PacBio sequencing, following the Arima low-input protocol using restriction enzymes *Dde*I and *Dpn*II. Proximity-ligated DNA was sheared using a Diagenode Bioruptor (Denville, New Jersey, USA) and a Swift Accel NGS 2S Plus kit (Integrated DNA Technologies, Coralville, Iowa, USA) was used to prepare Illumina sequencing libraries with insert sizes of 200 to 600 bp. An AVITI sequencer (Element Biosciences, San Diego, California, USA) was used to generate 150 bp paired-end sequences for the Hi-C libraries.

### Genome assembly, scaffolding, and quality control

We assembled the *B. fervidus* and *B. flavifrons* genomes using the same workflow (see Sim 2024), except for adding a “purge_dups” step required for the diploid sample (see below). We filtered adapters from raw HiFi reads using default setting in HiFiAdapterFilt v. 0.2.3 (Sim et al. 2022) and performed genome assemblies using HiFiASM v. 0.16.1-r375 (Cheng et al. 2021), using the “–n-hap 1” specification. This preliminary contig assembly was assessed for quality and completeness using BlobToolKit v2.6.1 (Kumar et al. 2013; Laetsch and Blaxter 2017; Challis et al. 2020) and the Benchmark of Single-Copy Orthologs (BUSCOs) with the hymenoptera_odb10 gene set (Waterhouse et al. 2018; Manni et al. 2021). We used GenomeScope2 (Ranallo-Benavidez et al. 2020) to estimate genome size and coverage, specifying a ploidy of 1 for *B. fervidus* and a ploidy of 2 for *B. flavifrons*. We also assessed kmer frequencies using Merqury (Rhie et al. 2020). For the diploid *B. flavifrons* assembly, we purged duplicates using purge_dups v1.2.5 (Guan et al. 2020) to increase the accuracy of the principal and alternate haplotypes by separating duplicate regions that result from diploidy into each haplotype. With the Hi-C data, we scaffolded the contigs using “Yet Another Hi-C Scaffolding tool” (Zhou et al. 2023) and generated Hi-C contact maps using the Juicebox tool set. We then manually curated the scaffolded assemblies in Juicebox (Durand et al. 2016; Dudchenko et al. 2018) and converted the curated scaffold using the Juicebox “juicebox_assembly_converter.py” script. Following conversion of the scaffolded assembly, we re-ran Blobtools and BUSCO to assess assembly quality and identify taxonomic origin of scaffolds. Scaffolds were assigned to their closest taxon using BLAST (Altschul et al. 1997) and Diamond (Buchfink et al. 2015) searches to NCBI nt and UniProt databases, respectively, within BlobToolKit (Kumar et al. 2013; Laetsch and Blaxter 2017). We initially retained “no-hit” scaffolds as the lack of a match might reflect a shortcoming of the database, and we subsequently checked that all scaffolds assigned to chromosomes contained hymenopteran BUSCOs and were orthologous to other *Bombus* genomes using synteny analysis (see below). Following the MitoHiFi v2 workflow (Uliano-Silva et al. 2023), we identified and removed the mitochondrial genome from the scaffolded assemblies for both *B. flavifrons* and *B. fervidus*, using *Bombus longipennis* Friese (NCBI accession: NC_057952.1) as the reference mitogenome. Mitochondrial sequences and scaffolds not retained for the final assemblies are available on Figshare (Lozier 2025).

Because the taxa belong to cryptic species complexes, we used the MitoHiFi-detected mitogenomes to confirm visual identification by the expert sample collector (J.B.U.K.). For *B. flavifrons,* we downloaded sequences confirmed as *B. flavifrons* and from a phenotypically similar species *B. centralis* Cresson, as well as *B. melanopygus* Nylander and *B. vagans* Smith as outgroups from GenBank (PQ857055-PQ857165), which were generated using DNA barcoding of the *Cytochrome Oxidase I* (COI) region following methods detailed in Lozier et al. (2020) . For *B. fervidus*, we downloaded NCBI sequences from a study (Koch et al. 2018) that identified clear genetic separation of *B. fervidus* and *B. californicus* using COI (MH747967.1-MH748028.1), 16S rRNA (MH747905.1-MH747966.1), and 12S rRNA (MH747843.1-MH747904.1; note these accessions include one sample of *B. insularis* [Smith] and one sample of *B. weisi* Friese as outgroup sequences, which we also use here for consistency). We also downloaded a previously assembled mitochondrial genome for *B. fervidus* (BK063621.1) (Gonçalves et al. 2024). For each species set, we aligned the mitochondrial scaffold(s) identified by MitoHiFi to Genbank sequences using Geneious Prime 2025.1 (Biomatters, Auckland, NZ), clipped the alignment to retain sequence data represented across all samples, and concatenated the 3 *B. fervidus* mtDNA genes into a single alignment. Sequence similarity was visualized using a simple neighbor-joining tree (HKY substitution model) in Geneious for each taxon set to ensure that assembled mitochondrial sequences clustered with the appropriate species. Alignments are available on Figshare (Lozier 2025).

### Genome annotation and synteny analysis

Genome annotations were performed using the NCBI Eukaryotic Genome Annotation Pipeline software version 10.3 (Thibaud-Nissen et al. 2016) to generate RefSeq accessions. Details on the annotation process, including details on the RNA and protein evidence used, are provided on the NCBI Annotation Release pages for each species (*B. flavifrons*: RefSeq Accession: GCF_040668555.1, annotation GCF_040668555.1-RS_2024_10; *B. fervidus*: RefSeq Accession GCF_041682495.1, annotation GCF_041682495.1-RS_2025_03). Following annotation, BUSCO scores were recalculated in protein mode using the peptide sequence derived from the longest isoform for each gene.

To identify repetitive elements in the assembled genomes, we used RepeatModeler v2.0.6 (Flynn et al. 2020) and RepeatMasker 4.1.7 (Smit et al. 2013) as installed by the Dfam TETools container v1.9 (https://github.com/Dfam-consortium/TETools), alongside the associated tools RECON v1.08 (Bao and Eddy 2002), RepeatScout v1.0.7 (Price et al. 2005), TRF v4.09.1 (Benson 1999), RMBlast v2.14.1, UCSC genome browser utilities v413, LTRharvest v1.6.4 (Ellinghaus et al. 2008), MAFFT v7.7471 (Katoh et al. 2002), cd-hit v4.8.1 (Li and Godzik 2006), HMMER v3.4 (Eddy 2023), NINJA (Wheeler 2009), and LTR_retriever v 2.9.0 (Ou and Jiang 2018).The RepeatModeler pipeline was used to identify de novo transposable elements for each species, which were then combined with the Dfam 3.8 database partition 7 (dfam38-1_full.7.h5) (Storer et al. 2021) for Apidae (famdb.py -i Libraries families –format fasta_name –include-class-in-name –ancestors –descendants 7458 > Apidae-rm.fa) to create species-specific repeat libraries for RepeatMasker. The distribution of protein coding genes, noncoding RNA (ncRNAs), and repeat elements identified by repeat masker were plotted using the R v4.4.1 (R Core Team 2021) package CIRCLIZE (Gu et al. 2014). RepeatMasker analyses were also repeated using only the species-specific RepeatModeler libraries.

To further examine completeness of the assembled chromosomes, confirm large-scale orthology with other *Bombus* taxa, and in particular to examine similarity between the 19th chromosome detected in *B. fervidus* (see Results section) with that of its close relative *B. pensylvanicus* (Lozier et al. 2025), we evaluated the synteny of the genomes with each other and with a set of RefSeq-annotated chromosome-scale assembles for representatives of other subgenera using GENESPACE v1.4 (Lovell et al. 2022). As a comparison for riparian plots, we used genomes from species representing common North American bumble bee subgenera that were sequenced and assembled using comparable methods, including *B. huntii* (Koch et al. 2024 ) in subgenus *Pyrobombus*, *B. affinis* Cresson (Koch et al. 2023) in subgenus *Bombus* s.s., and *B. pensylvanicus* (Lozier et al. 2025) in subgenus *Thoracobombus*. For each genome, we obtained the protein FASTA (translated CDS) file and gene model predictions (GFF converted to BED using the parse_annotations function) from the respective annotations. The GENESPACE pipeline uses OrthoFinder 3.01.1b1 (Emms and Kelly 2015; Emms and Kelly 2019) to assign orthologous groups among the annotated species. To visualize the variation in chromosome structure, GENESPACE riverine plots were used to map syntenic blocks and rearrangements (eg gaps, inversions, translocations) among the genomes. Data sets and scripts used for analyses are available on Figshare (Lozier 2025).

## Results and discussion

For *B. flavifrons*, PacBio Sequel IIe sequencing generated 2.7 million reads and 25.8G bases of data (average ± SD read length of 9,514 ± 4,619 bp), and Element Biosciences Aviti sequencing of the Arima Hi-C library generated 115.5 million read pairs. For *B. fervidus*, PacBio sequencing generated 2.7 million reads and 30.3G bases of data (average ± SD read length of 11,211 ± 4,435 bp), and Element Biosciences Aviti sequencing of the Hi-C library generated 156.2 million read pairs. Genomescope2 profiles for the species are presented in Supplementary Figs. 1a and 2a in File 1. Interestingly, analysis of PacBio reads for *B. flavifrons* using the taxonomy analysis tool on SRA indicated that only ∼41% of reads belonged to bumble bees, with most of the remaining reads (∼53%) belonging to bacteria, largely *Arsenophonus* spp. *Arsenophonus* spp. are insect-associated symbionts occasionally observed as “non-core” bumble bee endosymbionts that occur alongside the core microbiota in abdominal tissues but can become dominant when present (Parmentier et al. 2018; Hammer et al. 2021; Hotchkiss et al. 2025), consistent with its prevalence in *B. flavifrons* reads. For *B. fervidus,* 77.5% of reads could be identified and nearly all of these (∼96%) were identified as *Bombus*-derived. Similarly, our analysis of Blobtools results from the scaffolded assembly for *B. flavifrons* revealed a substantial fraction of non-Arthropod scaffolds, with 303 taxonomic matches to protists (Euglenozoa), Proteobacteria (Gammaproteobacteria), plants (Streptophyta), Nematoda, and fungi (Ascomycota), accounting for around 40 MB of sequence (see Supplementary Fig. 1 in File 1). *B. fervidus* contained considerably fewer contaminants with 26 scaffolds (17 MB) having taxonomic matches to bacteria, Streptophyta, Nematoda, Ascomycota, and ciliates (see Supplementary Fig. 2 in File 1). These non-arthropod scaffolds were subsequently removed from the assemblies (FASTA formatted sequences and taxonomic assignments available on Figshare; Lozier 2025).

**Fig. 2.**
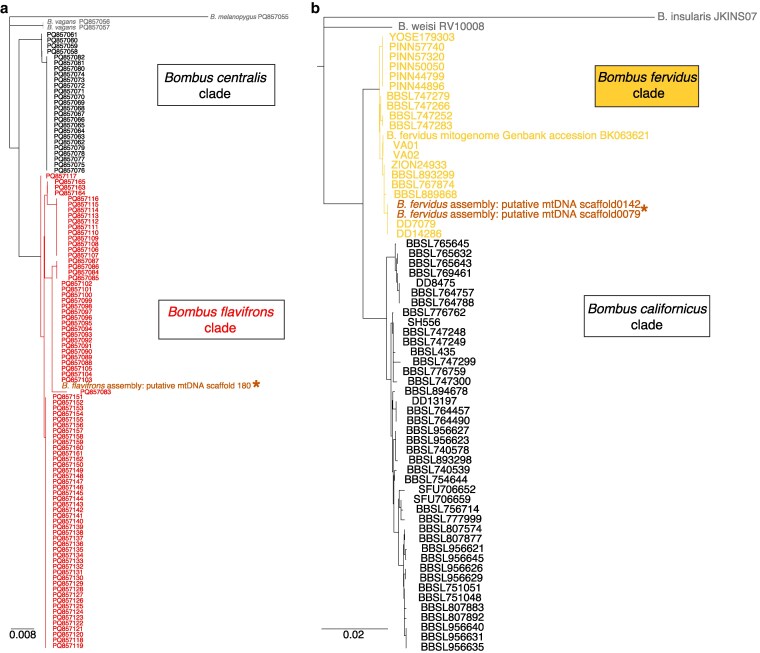
Neighbor-joining trees of assembled mitochondrial scaffolds and GenBank sequences from a) *B. flavifrons* and b) *B. fervidus* assemblies to confirm taxonomic status of sequenced specimens. For *B. flavifrons* the tree was constructed using XXXbp of COI sequences using the putative mitochondrial scaffold (scaffold180) and DNA barcode sequences from specimens confirmed to represent *B. flavifrons* and a cryptic codistributed species *B. centralis*. For *B. fervidus*, we included 1,187 concatenated bp of COI, 12S, and 16S regions for the 2 assembled mtDNA scaffolds from the *B. fervidus* assembly (identical except for a short indel in the noncoding AT-rich region), sequences from the study of Koch et al. that clarified the divergence between *B. fervidus* and *B. californicus*, and the homologous regions extracted from a previously published *B. fervidus* mitogenome (NCBI BK063621; Goncalves et al. 2024).

Because both species are cryptic and can be confused with other bumble bee taxa, we aligned the assembled mitochondrial scaffolds identified by the MitoHiFi workflow to mitochondrial sequences for relevant species obtained from GenBank. For *B. flavifrons*, MitoHiFi detected a single scaffold as mitochondrial, whereas 2 highly similar scaffolds were detected in *B. fervidus*, although these scaffolds were identical throughout protein coding regions, differing only by short indels in the noncoding AT-rich region between 12S rRNA and ND2 genes (sequences available on Figshare; Lozier 2025). Both genomes fell in appropriate clades in neighbor-joining trees (Fig. 2), with *B. flavifrons* clearly clustering with other *B. flavifrons* barcode sequences vs morphologically similar *B. centralis* (Fig. 2a), and *B. fervidus* mitochondrial sequences clustering with *B. fervidus* when compared with the sister species *B. californicus* (Fig. 2b).

After removing contaminating sequences, the final *B. flavifrons* assembly (iyBomFlav1_principal) was 310.5 Mb in length across 483 total scaffolds (scaffold N50 = 15.6 Mb, contig N50 = 13.5 Mb), with 18 major assembled scaffolds that correspond to the typical 18 chromosomes associated with most *Pyrobombus* species (length of top 18 = 270.94 Mb of 310.5 Mb total, range 6.4 to 23.1 Mb, scaffold coverage of ∼41x; Fig. 1; Supplementary Fig. 1 in File 1). The *B. fervidus* assembly (iyBomFerv1) was 314.3 Mb in length across 181 scaffolds (scaffold N50 = 15.3 Mb, contig N50 = 10.3 Mb), with 19 identified chromosomes (length of top 19 scaffolds = 280.5 Mb of 314.3 Mb total, range 4.6 to 26.6 Mb, scaffold coverage of ∼100x) (Fig. 1; Supplementary Fig. 2 in File 1). These genome sizes are in line with other comparable *Bombus* genomes (∼274 to 392 Mb in size) that have been assembled using some combination of long-read and chromatin capture sequencing (www.ncbi.nlm.nih.gov/datasets/genome/?taxon=28641, accessed 2025 July 21).

Annotation of *B. flavifrons* predicted 12,476 genes and pseudogenes (10,137 protein coding genes) and 3,893 ncRNAs (Table 1). The number of all genes identified is slightly lower, but similar to, other sequenced bumble bee genomes, especially for protein coding genes (see Koch et al. 2024 for comparison of several *Bombus* species). BUSCO analysis (v5.7.1) using the hymenoptera_odb10 data set indicated a complete genome and scores were comparable to other *Bombus* (Koch et al. 2024 ), with 97.9% of 5,991 benchmarking single-copy orthologs detected (97.6% single copy, 0.4% duplicated, 0.7% fragmented, 1.4% missing). Annotation of *B. fervidus* predicted 13,353 genes and pseudogenes (10,599 protein coding genes) and 3,853 ncRNAs (Table 1), which is similar to other bumble bees (Koch et al. 2024). BUSCO analysis identified 98.8% of single-copy orthologs in the assembly (98.3% single copy, 0.5% duplicated, 0.6% fragmented, 0.6% missing).

**Table 1.** Feature count summary of the *B. flavifrons* (iyBomFlav1_principal) and *B. fervidus* (iyBomFerv1) assemblies and annotations.

Feature	iyBomFlav1_principal	iyBomFerv1
Genes and pseudogenes	12,476	13,353
Protein coding	10,137	10,599
Noncoding	2,085	2,558
Transcribed pseudogenes	0	0
Nontranscribed pseudogenes	253	195
Genes with variants	5,318	5,256
Immunoglobulin/T-cell receptor gene segments	0	0
Other	1	1
mRNAs	23,981	24,003
Fully supported	23,176	23,439
With > 5% ab initio	496	310
Partial	47	48
With filled gap(s)	0	0
Known RefSeq (NM_)	0	0
Model RefSeq (XM_)	23,981	24,003
ncRNAs	3,893	3,853
Fully supported	3,543	2,862
With > 5% ab initio	0	0
Partial	0	2
With filled gap(s)	0	0
Known RefSeq (NR_)	0	0
Model RefSeq (XR_)	3,701	3,602
Pseudo transcripts	0	0
Fully supported	0	0
With > 5% ab initio	0	0
Partial	0	0
With filled gap(s)	0	0
Known RefSeq (NR_)	0	0
Model RefSeq (XR_)	0	0
CDSs	23,981	24,003
Fully supported	23,176	23,439
With > 5% ab initio	531	345
Partial	47	48
With major correction(s)	64	136
Known RefSeq (NP_)	0	0
Model RefSeq (XP_)	23,981	24,003

Repetitive content (Table 2) of the putative chromosomes was 31.51% in *B. flavifrons* and 33.31% in *B. fervidus* using the de novo RepeatModeler libraries for each species merged with the Dfam 3.8 Apidae repeat families library. For *B. flavifrons*, 6.44% of assembled chromosomes were identified as retrotransposons (mostly long interspersed nuclear elements, or LINEs, and long tandem repeats, or LTRs), 8.44% was identified as DNA transposons, 3.14% simple repeats, and 12.95% unclassified repeats. The detected repeat families were similar for *B. fervidus*, although *B. fervidus* had a slightly greater representation of DNA transposons, with 5.73% retrotransposons (again mostly LINEs and LTRs), 11.78% identified as DNA transposons, 1.35% simple repeats, and 13.89% unclassified repeats. The repeat percentages and classifications detected here correspond well with repeat content of putative chromosomes in other recent assemblies from these subgenera (Lozier et al. 2025). Repeating the RepeatMasker analyses using only the species-specific RepeatModeler libraries reduced the total masked repeat percentages detected for the species to 24.03% for *B. flavifrons* and 26.29% for *B. fervidus* (see Supplementary Table 1 in File 1).

**Table 2.** Summary of repeat elements in chromosomal scaffolds for *B. flavifrons* and *B. fervidus* assemblies from RepeatMasker run with custom libraries merging de novo RepeatModeler libraries for each species with models extracted for Apidae from the Dfam 3.8 FamDB partition 7 (taxon 7458).

		*B. flavifrons*			*B. fervidus*		
Element category		*N*	Length (bp)	% of c'somes	*N*	Length (bp)	% of c'somes
Retroelements		44,503	17,455,665	6.44	38,528	16,069,543	5.73
SINEs:		100	11,433	0.00	217	22,976	0.01
Penelope:		868	82,513	0.03	919	85,956	0.03
LINEs:		18,089	6,982,456	2.58	16,378	6,492,732	2.31
	CRE/SLACS	0	0	0.00	0	0	0.00
	L2/CR1/Rex	1,168	228,643	0.08	1,137	225,024	0.08
	R1/LOA/Jockey	6,815	3,174,826	1.17	6,279	2,817,082	1.00
	R2/R4/NeSL	511	319,463	0.12	496	240,561	0.09
	RTE/Bov-B	876	317,762	0.12	635	147,019	0.05
	L1/CIN4	275	29,170	0.01	256	29,584	0.01
LTR elements:		25,314	10,461,776	3.86	21,933	9,553,835	3.41
	BEL/Pao	2,086	791,260	0.29	2,389	1,217,912	0.43
	Ty1/Copia	3,447	1,152,285	0.43	2,870	714,445	0.25
	Gypsy/DIRS1	17,130	7,648,770	2.82	14,417	6,905,173	2.46
Retroviral		653	92,517	0.03	633	75,786	0.03
							
DNA transposons		65,085	22,855,993	8.44	65,157	33,039,980	11.78
	hobo-Activator	7,286	962,529	0.36	7,777	3,446,813	1.23
	Tc1-IS630-Pogo	37,785	6,343,139	2.34	37,301	6,359,808	2.27
	En-Spm	0	0	0.00	0	0	0.00
	MULE-MuDR	1,242	11,304,172	4.17	1,665	18,884,585	6.73
	PiggyBac	8,718	2,148,295	0.79	8,804	2,238,151	0.80
	Tourist/Harbinger	546	77,434	0.03	542	77,918	0.03
	Other	229	47,041	0.02	220	44,594	0.02
							
Rolling-circles		2,032	396,736	0.15	2,119	435,721	0.16
Unclassified		205,774	35,087,794	12.95	214,763	38,969,159	13.89
Total interspersed repeats:			75,481,965	27.86		88,164,638	31.44
							
							
Small RNA:		912	122,462	0.05	1,142	122,333	0.04
							
Satellites:		472	67,824	0.03	464	69,764	0.02
Simple repeats:		74,191	8,514,064	3.14	80,206	3,793,199	1.35
Low complexity:		15,229	779,336	0.29	16,354	836,800	0.30

RepeatMasker analyses run only using the RepeatModeler libraries can be found in Supplementary Table 1 in File 1.

**Fig. 3.**
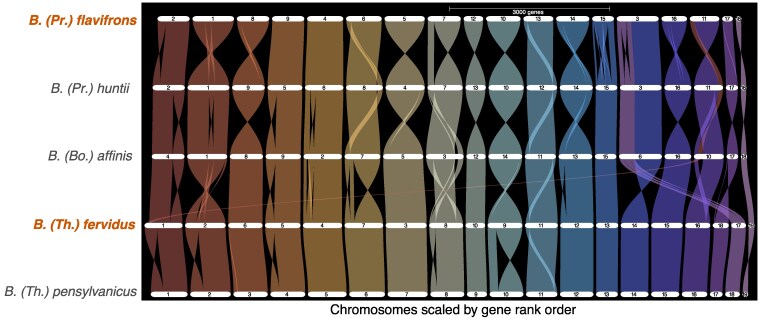
A GENESPACE riparian plot synteny map (top to bottom) of the 2 newly assembled genomes for *B. (Pyrobombus) flavifrons* and *B. (Thoracobombus) fervidus* with other North American species *B. (Pyrobombus) huntii, B. (Thoracobombus) pensylvanicus*, and *B. (Bombus) affinis*. The plot is organized according the 19 *B. pensylvanicus* pseudochromosomes (largest to smallest in physical size), with colored braids representing syntenic blocks between chromosomes the other bee genomes. Note that some chromosomes are assembled in opposite directions from the *B. pensylvanicus* reference (eg Chromosome 8 or 5 in *B. flavifrons* relative to orthologs in other taxa) such that whole chromosome braids do not always entirely indicate intrachromosomal rearrangements.

Riparian plots produced by GENESPACE showed high levels of overall synteny across the analyzed genomes, as generally observed in bumble bees with high quality assemblies (Sun et al. 2021; Koch et al. 2024). OrthoFinder identified and aligned 10,180 orthogroups (9,008 with all species present and 8,743 single copy) between *B. flavirons, B. fervidus, B. huntii, B. affinis*, and *B. pensylvanicus* that were used for synteny analysis (Fig. 3). Most observed rearrangements were intra-chromosome changes, especially near the ends of chromosomes. The only major rearrangements were the extra *Thoracobombus* chromosomes (chromosome 17 by rank size in *B. fervidus* and chromosome 18 in *B. pensylvanicus*) that resulted in a haploid chromosome number of 19 vs the more typical 18 observed in the other subgenera (Fig. 3). The extra chromosomes are homologous with each other and to the termini of longer chromosomes in the other species (chromosome 3 ranked by size in *B. flavifrons* and *B. huntii* and chromosome 6 by size in *B. affinis*, respectively). Interestingly, karyotyping of *B. fervidus* from eastern Canada has previously suggested that this species had 18 chromosomes (Owen et al. 1995), suggesting some potential for incomplete or misassembly, or alternatively additional chromosomal variation not discovered in karyotyping. However, this discrepancy would be worth further investigation with assemblies from additional specimens representing other parts of the *B. fervidus* geographic range, as karyotyping of the *B. fervidus* sister species *B. californicus* identified 19 chromosomes (Owen et al. 1995). The homology of the extra chromosome in the 2 North American *Thoracobombus* indicates either that these related species share a chromosome rearrangement that is not found in all members of the subgenus, including other New World species like *B. dahlbomii* Guérin-Méneville (Martínez et al. 2024), or that the taxa share a common feature that promotes misassembly of this region. Assembly of additional taxa from the sub-clade of *Thoracobombus* that contains *B. fervidus* and *B. pensylvanicus* (Cameron et al. 2007 ) would help refine our understanding of the evolution of this region of the genome.

In conclusion, we have provided 2 new reference-quality chromosome-scale genome assemblies for North American bumble bees that belong to 2 distantly related subgenera. These 2 genomes will be especially useful as references for phenotypically polymorphic species that belong to Müllerian mimicry complexes in North America and will thus provide new resources for uncovering the genetic basis of color pattern variation in bumble bees. Further, *B. fervidus* belongs to a lineage of bumble bees that has shown evidence of declining populations, including the closely related *B. pensylvanicus* and the South American species *B. dahlbomii*, and is itself considered as vulnerable by the IUCN (Hatfield et al. 2015). These genomes will also be of value for comparative conservation genomics to assess possible genetic differences among lineages and species that have suffered declines vs those that have remained relatively stable (eg Lozier 2014) as well as for whole genome phylogeographic studies of these species that could improve on those with traditional genetic markers (Koch et al. 2018; Sakulich et al. 2025 ) and uncover regions potentially involved in local adaptation (eg Heraghty et al. 2022). These new assemblies add to the growing number of bumble bee reference genomes and will contribute to future studies of diverse aspects of genome biology, ecology, and evolution in this charismatic insect group.

## Supplementary Material

jkag041_Supplementary_Data

## Data Availability

PacBio long-read genomic sequencing reads are available at the National Center for Biotechnology Information Sequence Read Archive (SRA) SRX23666152 and SRX23839126. Element Biosciences Aviti short-read Hi-C sequencing reads are available on SRA at SRX23666153 and SRX23839127. Annotations and assemblies are available on NCBI RefSeq GCF_040668555.1 and GCF_041682495.2. Scripts to assemble the genomes use those available on Ag Data Commons (https://doi.org/10.15482/USDA.ADC/25762431.v1; Sim 2024). Mitochondrial sequences and alignments, information on non-target scaffolds removed from the final assemblies, code for synteny plots, and scripts and outputs for RepeatModeler and RepeatMasker, are available on FigShare doi: 10.6084/m9.figshare.29840165. Supplemental material available at G3 online.
