# Supplementary material for: Reference genome assemblies for the North American bumble bees Bombus flavifrons and Bombus fervidus, two phenotypically polymorphic species from distinct phylogenetic lineages
Source: G3 (Bethesda). 2026 Feb 17;16(5):jkag041. doi: 10.1093/g3journal/jkag041 (PMC13148389; doi:10.1093/g3journal/jkag041)
Supplement: jkag041_Supplementary_Data [file jkag041_supplementary_data.docx]

**File S1: Supplementary Figures and Tables for “Reference genome assemblies for the North American bumble bees *Bombus flavifrons* and *Bombus fervidus*, two phenotypically polymorphic species from distinct phylogenetic lineages”**


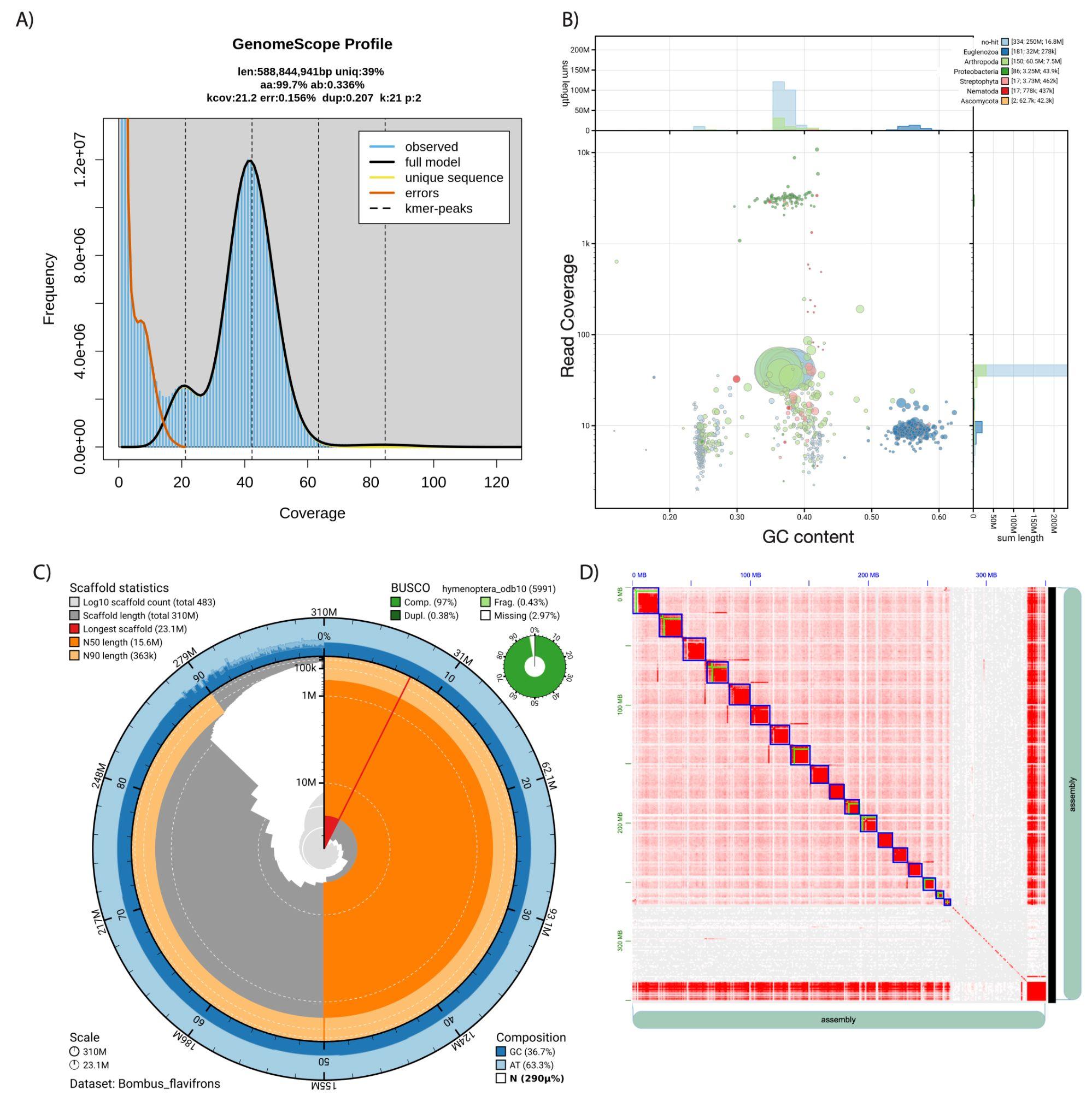


**Supplementary Figure S1.** Genome assembly details for *Bombus flavifrons*. A) Genomescope2 plot demonstrating high kmer coverage and low error rate, assuming a diploid genome. B) Blob plot for scaffolded assembly including non-Arthropod contaminants. C) Snail plot showing quality metrics for final scaffolded assembly (note Blobtoolkit BUSCO scores differ slightly than the standalone BUSCO v5.7.1 statistics reported in the main text). D) Juicebox Hi-C contact heatmap after manual curation showing scaffolding of contigs (green outlines) into 18 chromosomes (largest outlined blue boxes) and the unplaced scaffolds. Red color indicates interaction frequency between regions of DNA that are physically close to one another (x- and y-axis labels show the approximate size of the assembly in MB).

.
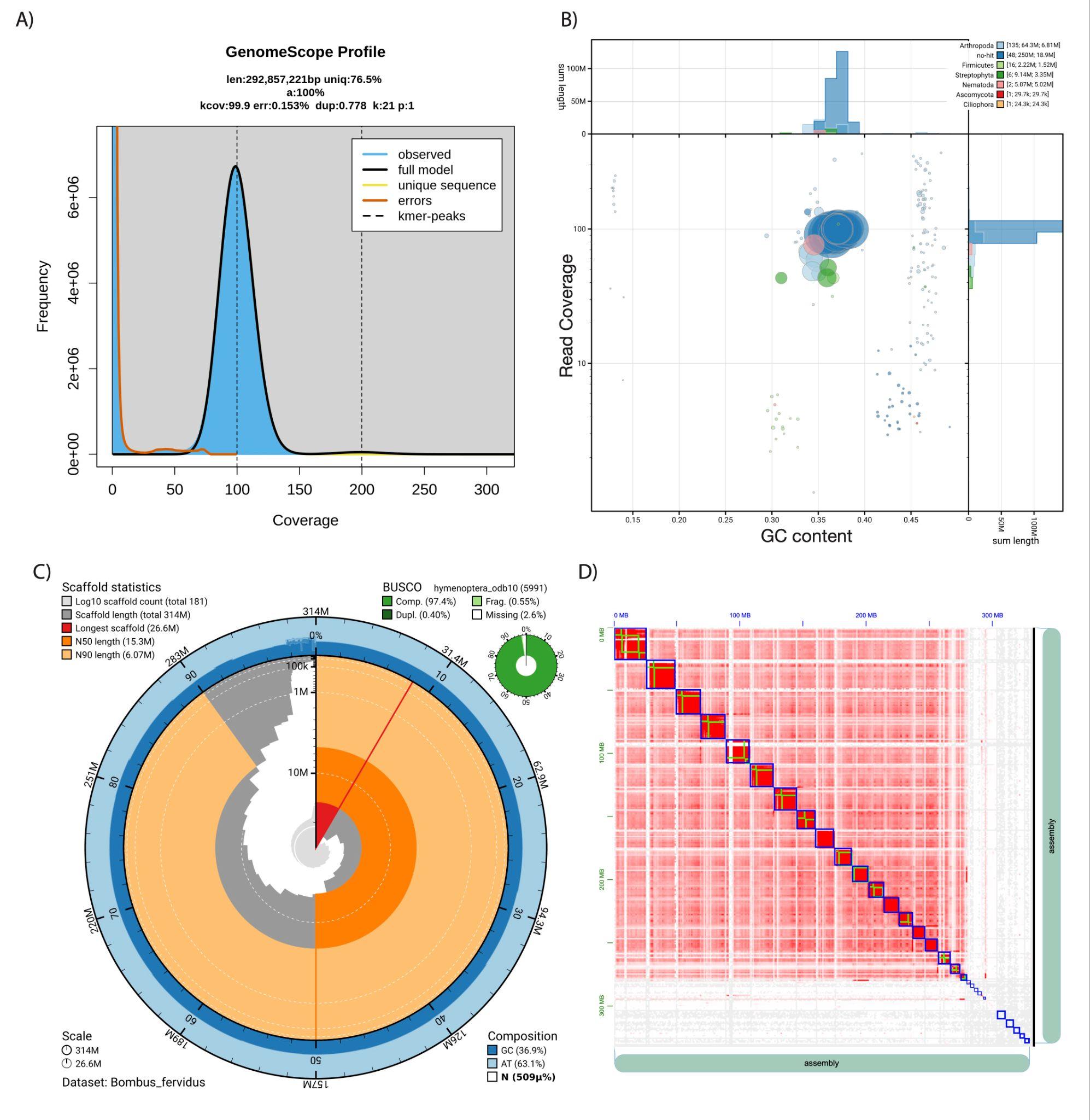


**Supplementary Figure S2.** Genome assembly details for *Bombus fervidus*. A) Genomescope2 plot demonstrating high kmer coverage and low error rate, assuming a haploid genome. B) Blob plot for scaffolded assembly including non-Arthropod contaminants. C) Snail plot showing quality metrics for final scaffolded assembly (note Blobtoolkit BUSCO scores differ slightly than the standalone BUSCO v5.7.1 statistics reported in the main text). D) Juicebox Hi-C contact heatmap after manual curation showing scaffolding of contigs (green outlines) into 19 chromosomes (largest outlined blue boxes) and the unplaced scaffolds. Red color indicates interaction frequency between regions of DNA that are physically close to one another (x- and y-axis labels show the approximate size of the assembly in MB).

**Supplementary Table S1.** Summary of repeat elements in chromosomal scaffolds for *B. flavifrons* and *B. fervidus* assemblies from RepeatMasker run with custom *de novo* RepeatModeler libraries for each species (excluding uncurated Apidae dfam repeat families).

|  |  | ***B. flavifrons*** | | |  | ***B. fervidus*** | | |
| --- | --- | --- | --- | --- | --- | --- | --- | --- |
| **Element Category** |  | **N** | **Length (bp)** | **% of chrom** |  | **N** | **Length (bp)** | **% of chrom** |
| **Retroelements** |  | 22,802 | 11,674,739 | 4.31 |  | 13,338 | 8,433,224 | 3.01 |
| SINEs: |  | 78 | 9,677 | 0.00 |  | 184 | 17,741 | 0.01 |
| Penelope: |  | 9 | 2,309 | 0.00 |  | 0 | 0 | 0.00 |
| LINEs: |  | 8,009 | 5,010,205 | 1.85 |  | 5,801 | 4,325,500 | 1.54 |
|  | CRE/SLACS | 0 | 0 | 0.00 |  | 0 | 0 | 0.00 |
|  | L2/CR1/Rex | 513 | 85,279 | 0.03 |  | 205 | 76,674 | 0.03 |
|  | R1/LOA/Jockey | 2,643 | 2,471,616 | 0.91 |  | 1,634 | 1,553,489 | 0.55 |
|  | R2/R4/NeSL | 191 | 285,274 | 0.11 |  | 147 | 153,684 | 0.05 |
|  | RTE/Bov-B | 513 | 197,861 | 0.07 |  | 205 | 64,293 | 0.02 |
|  | L1/CIN4 | 0 | 0 | 0.00 |  | 0 | 0 | 0.00 |
| LTR elements: |  | 14,715 | 6,654,857 | 2.46 |  | 7,353 | 4,089,983 | 1.46 |
|  | BEL/Pao | 211 | 361,112 | 0.13 |  | 1,487 | 1,052,610 | 0.38 |
|  | Ty1/Copia | 1,123 | 600,404 | 0.22 |  | 611 | 413,877 | 0.15 |
|  | Gypsy/DIRS1 | 2,718 | 2,863,765 | 1.06 |  | 1,640 | 1,424,255 | 0.51 |
| Retroviral |  | 0 | 0 | 0.00 |  | 30 | 27,207 | 0.01 |
|  |  |  |  |  |  |  |  |  |
| **DNA transposons** |  | 74,842 | 17,576,233 | 6.49 |  | 12735 | 12,652,073 | 4.51 |
|  | hobo-Activator | 2,691 | 1,348,124 | 0.50 |  | 1037 | 9,853,407 | 3.51 |
|  | Tc1-IS630-Pogo | 7,666 | 1,906,646 | 0.70 |  | 7715 | 1,637,641 | 0.58 |
|  | En-Spm | 0 | 0 | 0.00 |  | 0 | 0 | 0.00 |
|  | MULE-MuDR | 0 | 0 | 0.00 |  | 0 | 0 | 0.00 |
|  | PiggyBac | 9,154 | 1,631,213 | 0.60 |  | 2663 | 813,847 | 0.29 |
|  | Tourist/Harbinger | 74 | 15,802 | 0.01 |  | 120 | 19070 | 0.01 |
|  | Other | 0 | 0 | 0.00 |  | 0 | 0 | 0.00 |
|  |  |  |  |  |  |  |  |  |
| **Rolling-circles** |  | 135 | 44,684 | 0.02 |  | 484 | 158,125 | 0.06 |
| **Unclassified** |  | 127,827 | 25,729,899 | 9.50 |  | 141,419 | 47,133,219 | 16.81 |
| **Total interspersed repeats:** |  |  | 54,983,180 | 20.29 |  |  | 68,218,516 | 24.32 |
|  |  |  |  |  |  |  |  |  |
|  |  |  |  |  |  |  |  |  |
| **Small RNA:** |  | 0 | 0 | 0.00 |  | 184 | 17,741 | 0.01 |
|  |  |  |  |  |  |  |  |  |
| **Satellites:** |  | 0 | 0 | 0.00 |  | 9 | 2,385 | 0.00 |
| **Simple repeats:** |  | 87,342 | 9,157,827 | 3.38 |  | 92,737 | 4,394,882 | 1.57 |
| **Low complexity:** |  | 17,790 | 919,909 | 0.34 |  | 18,726 | 970,760 | 0.35 |
